# Efficacy and safety of oral Chinese patent medicine for benign prostatic hyperplasia: a network meta-analysis of randomized controlled trials

**DOI:** 10.3389/fmed.2024.1483864

**Published:** 2024-12-27

**Authors:** Yiwen Tang, Yuyang Cai, Jiasen Ding, Ronfu Ji, Xiong Wang, Zejia Zhang, Feng Xu, Zhan Gao

**Affiliations:** ^1^Graduate School of Beijing University of Chinese Medicine, Beijing, China; ^2^Xiyuan Hospital, China Academy of Chinese Medical Sciences, Beijing, China

**Keywords:** network meta-analysis, benign prostatic hyperplasia, oral Chinese patent medicines, efficacy, safety

## Abstract

**Objective:**

This paper aims to evaluate the disparities in efficacy and safety across various oral Chinese patent medicines for the treatment of benign prostatic hyperplasia (BPH), using a frequency-based reticulated meta-analysis.

**Methods:**

The researchers searched the following databases: Web of Science, PubMed, Excerpta Medical Database (Embase), Cochrane Library, China Knowledge Network (CNKI), China Biomedical Literature Service System (SinoMed), Wanfang Data Knowledge Service Platform and China Science and Technology Periodicals Database (VIP). Besides, the researchers collected all randomized controlled trials (RCTs) of oral Chinese patent medicines, as well as simple preparations and simple preparations for benign prostatic hyperplasia from the establishment of the database until July1, 2024. After two researchers independently screened literature, extracted data, and evaluated the risk of bias in the included studies, a net meta-analysis was conducted using Stata 16.0 software.

**Results:**

Seventy-two RCTs involving 15 oral Chinese patent medicines and a total of 7,800 patients were included. Net meta-analysis manifested that “Jinkuishenqi capsule (JKSQ) + conventional western medicine (CWM)” was the most effective way in increasing total efficiency ratio. “Huange capsule (HE) + CWM” was the most effective method in decreasing prostate volume. “Qianliesutong capsule (QLST) + CWM” was the most effective approach in decreasing residure volume. “Xialiqi capsule (XLQ) + CWM” was the most effective way in increasing maximum urinary flow rate. “Longbisu capsule (LBS) + CWM” was the most effective method in decreasing international prostate symptom score (IPSS). To reduce the adverse reactions, “HE + CWM” has the best efficacy. Considering both drug efficacy and safety, “Ningmitai capsule (NMT) + CWM” would be the most ideal choice.

**Conclusion:**

Based on NMA, JKSQ, HE, QLST, XLQ, LBS, NMT plus CWM have been proved to possess the highest probability of being the best therapy. Due to the limitations of this study, these results should be confirmed through detailed randomized controlled trials.

**Systematic review registration:**

https://www.crd.york.ac.uk/prospero/, Identifier, CRD42023484071.

## 1 Introduction

Benign prostatic hyperplasia (BPH) is a prevalent condition among middle-aged and elderly men, characterized by urinary frequency, urgency, and progressive dysuria, invariably associated with bladder outlet obstruction, significantly impacting the quality of the patients’ life in this demographic. For older men, the aging process and the presence of functional testes result in hormonal imbalances and altered cellular processes, causing hyperplasia of the prostate’s interstitial glandular components and prostatic hypertrophy (1). Studies have shown that the prevalence of prostatic hyperplasia in men over the age of 45 reaches 45% in the United States and 80% by the age of 70 (2, 3) and it increases with age, risk factors including diet, exercise, smoking, and inflammation (4), and that the number of people with prostatic hyperplasia has reached 205 million globally (5), with an increasing burden of diseases (6). However, the efficacy of *α*-blockers (e.g., doxazosin) and 5αreductase inhibitors (e.g., finasteride) has been controversial, and the use of the drugs is limited by adverse effects such as the production of upright hypotension and erectile dysfunction. Therefore, the researchers need to choose the drug with fewer side effects and a longer course of treatment due to the longer course of the treatment (7).

**Figure 1:**
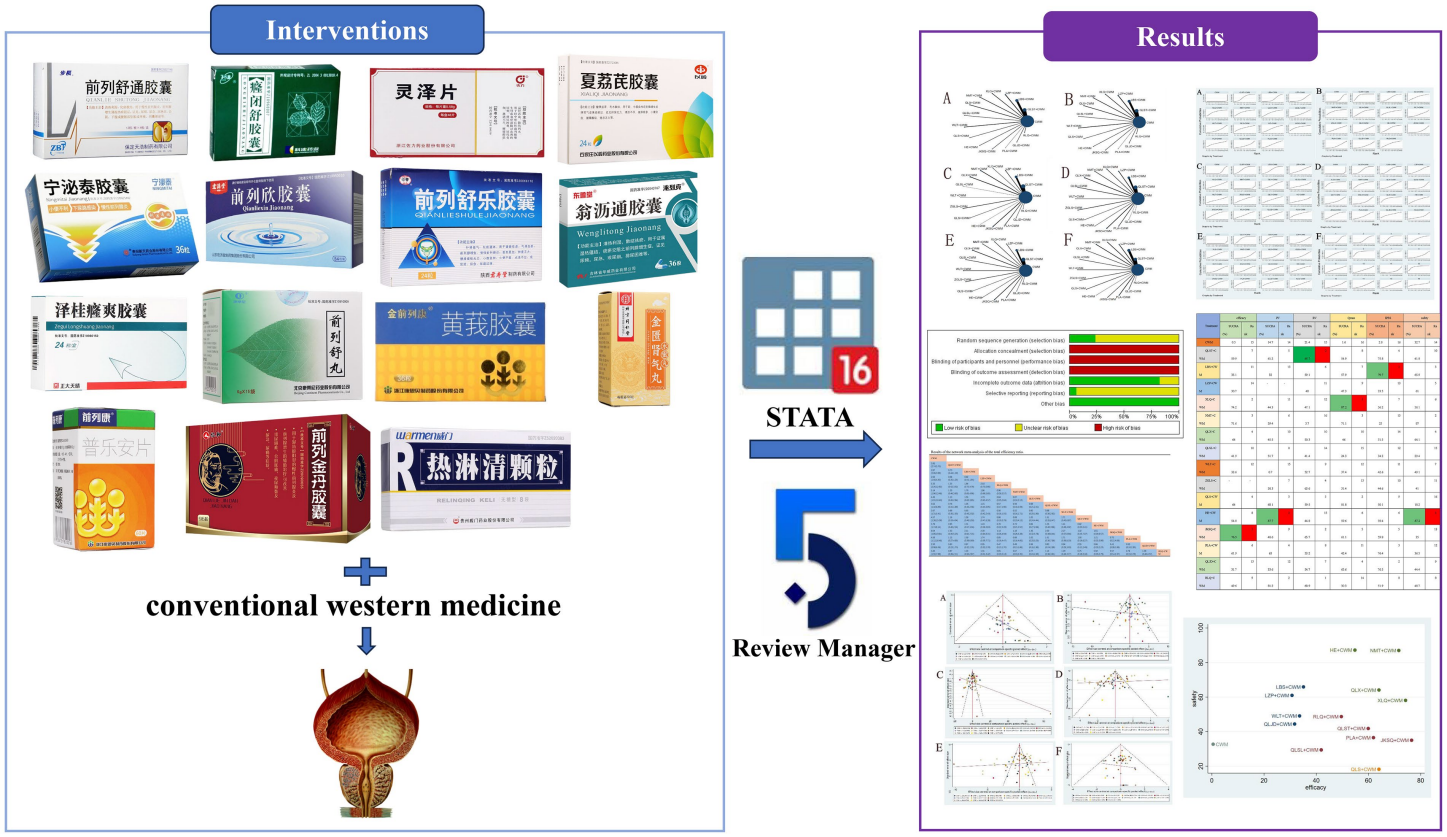
Graphic workflow for the NMA.

According to traditional Chinese medicine theory, BPH is referred to as “Jinglong” and “Longbi.” Clinical practice has shown that Chinese medicine has great advantages in the treatment of BPH, and oral Chinese patent medicines (OCPMs) have been widely used in the clinic due to their multi-targeted effects, simplicity of use, and low adverse effects. However, there are many types of OCPMs and there is a lack of cross-sectional comparisons (8). Network meta-analysis (NMA) combines the available evidence and allows for simultaneous comparisons of different therapeutic options. Therefore, this study was based on a network meta-analysis based on a frequency-based framework to compare the efficacy and safety of 15 different OCPMs to reveal the optimal OCPM for BPH treatment and to illustrate more perspectives on the choice of medicine for BPH. The graphic workflow in our research is illustrated in Figure 1.

## 2 Methods

### 2.1 Eligibility criteria

#### 2.1.1 Types of studies

Randomized controlled trial (RCT) studies, limited to Chinese and English studies, with no restriction on the blinding method used for the trial or the publication platform.

#### 2.1.2 Types of participants

Patients with benign prostatic hyperplasia meet the relevant criteria in the Chinese Urology and Male Diseases Diagnostic and Treatment Guidelines 2022 Edition, i.e.: ① men over 50 years old, with the following urinary symptoms: with urinary frequency, urinary urgency, increased nocturia, difficulty in urination, incontinence of urination, post urinary dribbling, etc.; ② rectal fingerprinting: increase in the size of the prostate gland, with the central sulcus becoming shallower or disappearing; ③ ultrasound: prostate gland volume > 20 mL; Urine flow rate examination: urine volume > 150 mL, maximum urine flow rate < 15 mL/s; ⑤ International Prostate Symptom Score (IPSS) >5 points and ≤ 19 points. BPH can be diagnosed if more than 2 of the above symptoms and auxiliary tests are met.

#### 2.1.3 Types of interventions

The experimental group used OCPM combined with conventional CWM; the control group used CWM alone or placebo combined with CWM.CWM included *α*-blockers (e.g., doxazosin mesylate, terazosin hydrochloride, tamsulosin hydrochloride), 5α-reductase inhibitors (e.g., finasteride, eplerenone), M-receptor antagonists (e.g., tolterodine tartrate), and treatment regimens consisting of a combination of the above drugs, for the convenience of the performing reticulated Meta-analysis as well as to simplify the analyzed data. They were uniformly named as CWM.

#### 2.1.4 Types of outcomes

The primary outcome was the total efficiency ratio. The secondary outcome were prostate volume, residure volume, maximum urinary flow rate and IPSS. The safety indexes were the adverse reactions occurred during the study observation. The total effective rate = (apparent effect + effective)/total number of cases × 100%.

### 2.2 Excluded criteria

(1) Studies that do not explicitly include patients with benign prostatic hyperplasia or exclude patients with other complications; (2) studies in which the intervention involved unlisted OCPM preparations in hospitals, combination of other TCM therapies in addition to OCPMs, or combination of both or more OCPMs; (3) studies of the type of dissertation, animal experiments, clinical trial protocols, and self-control trials; (4) studies with missing data, unavailable full text, duplicate publications, or incorrect data; (5) literature of OCPM studies with less than 2 RCTs; (6) studies with a sample size of less than 20 cases in trial groups.

### 2.3 Search strategy

We searched Web of Science, PubMed, Cochrane Library, China Knowledge Network, Wanfang Data and Wipu databases, and the search time was from the establishment of the database until July 1, 2024, and the search languages were Chinese and English. The search was conducted by combining subject words and free words, and the search strategy was developed according to different databases. The English search terms included: benign prostatic hyperplasia, oral Chinese patent medicines, Chinese herbal drugs, International English search terms include: benign prostatic hyperplasia, oral Chinese patent medicines, Chinese herbal drugs, International Prostate Symptom Score, Postvoid Residure volume, Prostate Volume, mean maximal flow rate, randomized controlled trial, RCT, etc. Chinese search terms include: prostatic hyperplasia, Chinese patent medicine, traditional Chinese medicine, tablet, bulk, capsule, capsule, tablets, bulk, capsules, pills, granules, effective rate, IPSS, bladder residual urine volume, prostate volume size, maximal flow rate, quality of life score, randomized controlled, trial, clinical, efficacy, and so on. References to the included literature were also traced for more relevant studies. The detailed search strategies are described in Supplementary File 1.

### 2.4 Study selection and data extraction

The results of the literature search were uploaded to NoteExpress, and after checking the weight of the software, two researchers screened independently according to the inclusion and exclusion criteria, and then, reading the full text of the literature obtained from the initial screening and rescreened, and if there was any disagreement, a third researcher took part to decide on the final inclusion of the literature. Data were extracted from the screened literature using Excel 2021, including: publication date, study title, first author, sample size, mean age, intervention, and outcome indicators.

### 2.5 Quality evaluation

The Cochrane Risk of Bias Assessment Tool provided by RevMan 5.4 software was used to assess the risk of bias of the included literature, which included 7 items of randomized sequence generation, allocation concealment, blinding of investigators and subjects, blinding of outcome assessors, incomplete outcome data, selective reporting, and other biases, and the answers were provided for each item according to the categories of “low risk,” “unclear,” “high risk,” and any disagreements were resolved through discussion and consultation with the third investigator (9).

### 2.6 Statistical analysis

For categorical variables, the ratio of ratios (OR) was used, and continuous variables were expressed by applying the mean difference (MD) and its 95% confidence interval (CI). Traditional Meta-analysis was performed using RevMan 5.4 software, and inter-study heterogeneity was assessed using chi-square test and *I^2^* values. If there was no significant inter-study heterogeneity (*p* < 0.05, *I^2^* < 50%), the analysis was performed using a fixed-effects model. Conversely, the analysis was performed using a random-effects model. If inter-study heterogeneity was excessive (*p* < 0.01 or *I^2^* >50%), descriptive analysis was used.

Meta-analysis was performed based on the frequency-based framework using the network and mvmeta packages of Stata 16.0 software to visualize the network diagrams of the evidence between the outcome indicators and the interventions to calculate the surface under the cumulative ranking curve (SUCRA), and to perform a “comparison-correction” funnel plot to identify any small-sample effect or publication bias. The area under the cumulative ranking curve (SUCRA) was calculated and ranked to compare the efficacy of the interventions in each outcome indicator, and a “comparison-correction” funnel plot was drawn to identify whether there was a small-sample effect or publication bias. When studies had closed loops, inconsistency tests were performed to assess the degree of agreement between the results of direct and indirect comparisons.

## 3 Results

### 3.1 Selection and identification of studies

**Figure 2:**
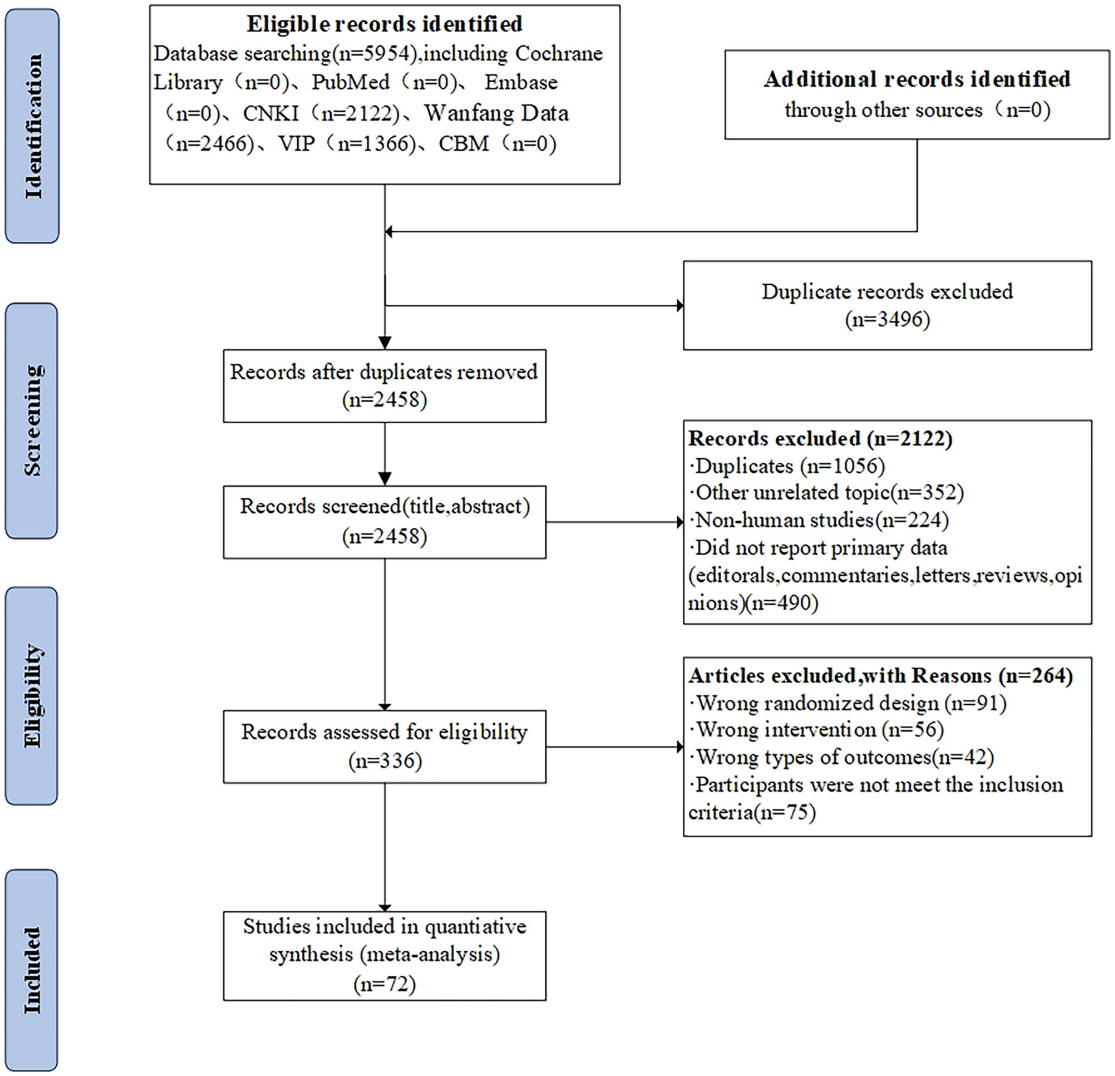
PRISMA flow diagram for eligible RCTs.

The title and abstract of 2,122 studies that did not meet the predefined inclusion criteria were excluded, resulting in 5954 citations, including 3,496 duplicate studies, and 336 full-text articles. A total of 336 full-text articles were screened and 264 were excluded due to inappropriate study designs, interventions, study objects, or outcomes. In total, 72 two-armed RCTs were included, which were conducted in China and published between 2009 and 2023. The PRISMA flow diagram is depicted in Figure 2.

### 3.2 Study characteristics of the involved researches

Seventy-two trials (10–81) involving 7,800 patients with BPH were included. 3,915 patients in the experimental group were treated with one of 15 proprietary Chinese medicines (Table 1) in combination with conventional western medicines, and 3,885 patients in the control group were treated with CWM alone.

**Table 1:** Basic characteristics of the included studies.

Study ID	Sample size/experiment/control	Age(Mean ± SD)		Intervention		Duration (weeks)	Outcomes
		Experiment	Control	Experiment	Control		
Mai et al. (2023) (10)	43/43	61.37 ± 2.52	62.34 ± 2.25	QLST+CWM	CWM	12	①②③④⑤
Gao et al. (2022) (11)	45/45	66.14 ± 3.60	65.03 ± 3.87	QLST+CWM	CWM	12	②③④⑤⑥
Li et al. (2020) (12)	64/64	63.4 ± 4.8	63.7 ± 4.2	QLST+CWM	CWM	8	①②③④⑤⑥
Tong et al. (2020) (13)	40/40	62.32 ± 5.01	62.23 ± 4.51	QLST+CWM	CWM	12	①②③⑤⑥
Kong et al. (2019) (14)	64/64	55.6 ± 2.1	55.5 ± 2.2	QLST+CWM	CWM	12	①②③④⑤⑥
Yin et al. (2018) (15)	53/53	66.4 ± 3.0	67.6 ± 2.2	QLST+CWM	CWM	12	①②③④⑤
Wang and Yu (2018) (16)	61/61	64.3 ± 2.5	63.9 ± 2.4	QLST+CWM	CWM	12	①②③④⑤
Liang et al. (2018) (17)	60/60	62.30 ± 2.14	62.25 ± 2.11	QLST+CWM	CWM	8	①②③④⑤⑥
Tian et al. (2018) (18)	45/45	71.08 ± 5.54	70.20 ± 5.21	QLST+CWM	CWM	8	①②③④⑤⑥
Wan (2017) (19)	56/56	60.2 ± 2.1	60.5 ± 2.3	QLST+CWM	CWM	12	①②③④⑤⑥
Li (2017) (20)	43/43	59.2 ± 3.7	59.9 ± 3.9	QLST+CWM	CWM	12	①②③④⑤
Man et al. (2017) (21)	45/45	67.48 ± 1.35	67.44 ± 1.37	QLST+CWM	CWM	12	①②③④⑤⑥
Yu et al. (2016) (22)	48/48	60.21 ± 8.50	60.40 ± 8.26	QLST+CWM	CWM	12	①②③④⑤
Shi et al. (2016) (23)	44/44	65.36 ± 2.53	65.34 ± 2.51	QLST+CWM	CWM	8	①②③④⑤⑥
Li et al. (2015) (24)	54/60	59.0 ± 8.6	58.8 ± 9.4	QLST+CWM	CWM	12	①②③④⑤⑥
Fu et al. (2015) (25)	73/74	74.1 ± 10.8	73.3 ± 11.2	QLST+CWM	CWM	4	②③④⑤⑥
Xuan et al. (2012) (26)	40/40	63.4 ± 13.8	64.1 ± 15.2	QLST+CWM	CWM	4	①②③④⑤⑥
Ma et al. (2009) (27)	82/82	62.7 ± 21.8	63.1 ± 23.4	QLST+CWM	CWM	8	①④⑤⑥
Wang (2014) (28)	40/40			QLST+CWM	CWM	12	①②③④⑤
Zhang et al. (2022) (29)	53/53	63.5 ± 6.5	64.5 ± 6.5	LBS + CWM	CWM	4	①③④⑤⑥
Xu et al. (2021) (30)	31/31			LBS + CWM	CWM	4	①③④⑥
Lu et al. (2020) (31)	55/55	68.8 ± 3.5	69.2 ± 3.2	LBS + CWM	CWM	26	①②③④⑤⑥
Zhou et al. (2018) (32)	66/66	58.6 ± 8.7	59.5 ± 9.2	LBS + CWM	CWM	12	①②③④⑤⑥
Ji et al. (2018) (33)	108/108	62.98 ± 6.41	63.25 ± 6.47	LBS + CWM	CWM	16	①②③④⑤⑥
Chen et al. (2017) (34)	58/58	68.35 ± 3.11	68.26 ± 3.07	LBS + CWM	CWM	12	①②③④⑤⑥
Yuan (2017) (35)	47/47	68.29 ± 8.57	68.45 ± 8.46	LBS + CWM	CWM	12	①②③④⑤⑥
Meng (2017) (36)	35/35	75.36 ± 6.54	76.38 ± 2.89	LBS + CWM	CWM	12	①③④
Xue et al. (2017) (37)	58/58	76.58 ± 6.32	75.87 ± 5.79	LBS + CWM	CWM	12	①③④⑤⑥
Song et al. (2016) (38)	115/113	65.7 ± 7.7	64.8 ± 7.9	LBS + CWM	CWM	52	①②③④⑤⑥
Zhang (2016) (39)	46/46	69.8 ± 12.2	68.24 ± 12.5	LBS + CWM	CWM	12	①⑤⑥
Chang et al. (2015) (40)	30/30	65.61 ± 6.95	63.19 ± 8.08	LBS + CWM	CWM	24	①④⑤⑥
Niu et al. (2014) (41)	60/50	63.5	61.3	LBS + CWM	CWM	16	②③④⑤⑥
Zhu (2023) (42)	35/35	61.15 ± 10.83	60.11 ± 9.86	LZP + CWM	CWM	8	①③④⑥
Zhao et al. (2023) (43)	150/150	58.65 ± 7.94	58.67 ± 7.99	LZP + CWM	CWM	6	①③④⑤⑥
Wang et al. (2022) (44)	42/42	71.2 ± 8.7	71.9 ± 8.7	LZP + CWM	CWM	4	①④⑤
Li et al. (2022) (45)	160/160	59.17 ± 8.03	58.93 ± 8.47	LZP + CWM	CWM	12	①③④⑤⑥
Liang et al. (2020) (46)	28/28	62.9 ± 6.1	63.2 ± 5.5	XLQ + CWM	CWM	4	⑥
Luo and Feng (2020) (47)	40/40	65.2 ± 4.7	64.7 ± 5.0	XLQ + CWM	CWM	12	①②③④⑤⑥
Gong (2020) (48)	50/50	69.87 ± 3.6	70.1 ± 3.9	XLQ + CWM	CWM	4	①④⑤
Yang and Wang (2019) (49)	50/50	68.10 ± 6.09	68.13 ± 6.15	XLQ + CWM	CWM	12	①②③④⑤⑥
Li et al. (2020) (50)	44/44	62.18 ± 5.19	62.15 ± 5.27	NMT + CWM	CWM	12	①②③④⑤⑥
Zhang (2020) (51)	37/36	61.24 ± 3.43	62.33 ± 2.67	NMT + CWM	CWM	2	①③④⑤
Ye (2019) (52)	31/31	65.3 ± 5.3	67.9 ± 6.2	NMT + CWM	CWM	9	①②③④⑥
Deng et al. (2018) (53)	20/20			NMT + CWM	CWM	2	③⑤⑥
Zhao et al. (2020) (54)	121/121	65.62 ± 1.75	65.24 ± 1.36	QLX + CWM	CWM	4	①②③④⑤⑥
Xu et al. (2020) (55)	78/79	65.32 ± 3.86	66.14 ± 4.12	QLX + CWM	CWM	12	①②③④⑤⑥
Wang et al. (2017) (56)	48/46	65.58 ± 7.48	66.12 ± 6.87	QLX + CWM	CWM	12	①②③④⑤
Gao et al. (2011) (57)	33/30	71 ± 7	70 ± 6	QLX + CWM	CWM	4	②③④⑤
Fan et al. (2023) (58)	50/50	62.20 ± 6.46	62.84 ± 6.90	QLSL+CWM	CWM	12	①②③④⑤⑥
Ye et al. (2020) (59)	32/32	62.40 ± 10.40	61.95 ± 9.51	QLSL+CWM	CWM	12	①④⑤
Cui et al. (2020) (60)	33/33	59.58 ± 7.49	60.15 ± 5.76	QLSL+CWM	CWM	12	④⑤⑥
Gu (2021) (61)	48/48	64.57 ± 1.29	64.88 ± 1.51	WLT + CWM	CWM	12	①②③④⑤⑥
Jiang and Wu (2019) (62)	120/120	71.86 ± 7.59	72.84 ± 7.52	WLT + CWM	CWM	4	①②⑤⑥
Zhang and Cheng (2012) (63)	48/52	66.7 ± 5.5	65.8 ± 5.3	WLT + CWM	CWM	12	①②④⑤
Xiang and Xiao (2012) (64)	56/42	63.1	63.5	ZGLS+CWM	CWM	8	③④⑤⑥
Liu et al. (2009) (65)	42/42	64.5	64.5	ZGLS+CWM	CWM	12	③④⑤
Li et al. (2009) (66)	70/70	60.6 ± 7.5	60.1 ± 8.2	ZGLS+CWM	CWM	8	②③④⑤⑥
Zhang et al. (2020) (67)	50/50	65.51 ± 4.55	65.65 ± 6.61	QLSW+CWM	CWM	12	①②③④⑤⑥
Chang et al. (2020) (68)	40/40	63.63 ± 4.90	63.51 ± 4.84	QLSW+CWM	CWM	4	①②③④⑤⑥
Cheng (2023) (69)	50/50	62.18 ± 8.24	64.34 ± 7.21	HE+CWM	CWM	6	①②③④⑤
Sun et al. (2019) (70)	50/50	65.83 ± 5.83	66.43 ± 5.48	HE+CWM	CWM	6	①②④⑤⑥
Xu et al. (2021) (71)	30/30	68.88 ± 4.03	69.00 ± 4.10	JKSQ+CWM	CWM	8	①②③④⑤⑥
Zhi et al. (2020) (72)	33/33	64.15 ± 2.31	63.27 ± 1.45	JKSQ+CWM	CWM	24	②③④⑤
Shen (2018) (73)	43/43	69.2 ± 4.7	69.2 ± 4.7	JKSQ+CWM	CWM	12	②③④⑤⑥
Liao et al. (2016) (74)	42/43	69.29 ± 8.76	69.38 ± 8.95	JKSQ+CWM	CWM	24	①③④⑤
Kong et al. (2020) (75)	44/44	64.73 ± 3.52	64.85 ± 3.27	PLA + CWM	CWM	4	③④⑤⑥
Li (2012) (76)	67/66	65.2 ± 3.8	64.6 ± 4.1	PLA + CWM	CWM	4	②③④⑤⑥
Su (2012) (77)	32/32	61.6 ± 4.2	62.5 ± 4.1	PLA + CWM	CWM	8	②③④⑤⑥
Ma et al. (2020) (78)	67/66	68.24 ± 16.9	69.2 ± 18.65	QLJD+CWM	CWM	12	①②③④⑤⑥
Zhang (2014) (79)	30/30	78.83 ± 5.62	79.13 ± 4.84	QLJD+CWM	CWM	52	①②③⑤⑥
Luo (2013) (80)	60/60	62.84 ± 4.05	63.46 ± 4.17	RLQ + CWM	CWM	4	①②③④⑤
Yuan and Luo (2012) (81)	49/38			RLQ + CWM	CWM	4	①③⑤⑥

①: Total efficiency ratio; ②: prostate volume; ③: residure volume; ④: maximum urinary flow rate; ⑤: international prostate symptom score, IPSS; ⑥: adverse reactions.

Treatments ranged from 2 to 52 weeks in duration. Among these RCTs, the most frequently used were QLST (19 studies, 1,000 cases), LBS (13 studies, 762 cases), LZP (4 studies, 387 cases), XLQ (4 studies, 168 cases), NMT (4 studies, 132 cases), QLX (4 studies, 280 cases), QLSL (3 studies, 115 cases), WLT (3 studies, 216 cases), ZGLS (3 studies, 168 cases), QLSW (2 studies, 90 cases), HE (2 studies, 100 cases), JKSQ (4 studies, 148 cases), PLA (3 studies, 143 cases), QLJD (2 studies, 97 cases), RLQ (2 studies, 109 cases). Improvement of lower urinary tract symptoms, smooth muscle relaxation to relieve bladder outlet obstruction, and reduction of prostate volume were the primary therapeutic options for the treatment of all patients. The characteristics of the 72 eligible randomized controlled trials are shown in Table 1.

### 3.3 Methodological quality assessment

**Figure 3:**
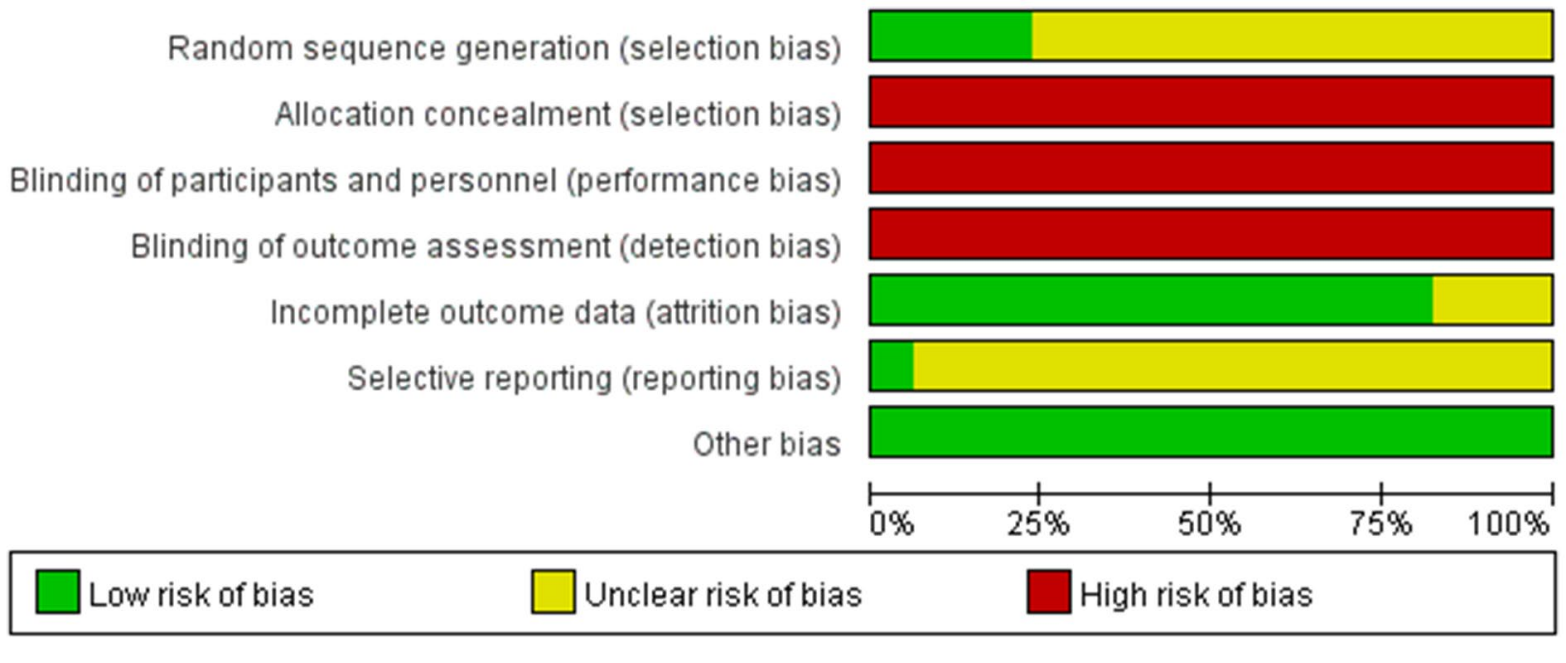
Risk of bias graph.

Two researchers separately evaluated the risk of bias of the included studies using the Cochrane Risk of bias tool presented in the Cochrane Handbook 5.1. The results of the assessment items were as follows: Low-risk items: (1) 15 studies in selective bias (interpretation using a random number table) and 3 studies on reporting bias (description using the shakedown method). (2) 62 studies in attrition bias (complete reporting of outcome data). (3) all studies in other bias (baseline of randomized controlled trials described). High-risk events: (1) All studies in selection bias (allocation concealment not used). (2) All studies in performance bias (blinding of participants and personnel not used). (3) All studies in detection bias (blinding of outcome assessments not reported). Risk items were unclear: (1) 57 studies in selection bias (specific randomization method not stated) and 69 studies in reporting bias (unclear selective reporting). (2) 10 studies in attrition bias (unclear selective reporting). A detailed description of the risk of bias assessment is shown in Figure 3.

### 3.4 Outcomes

#### 3.4.1 Total efficiency ratio

**Figure 4:**
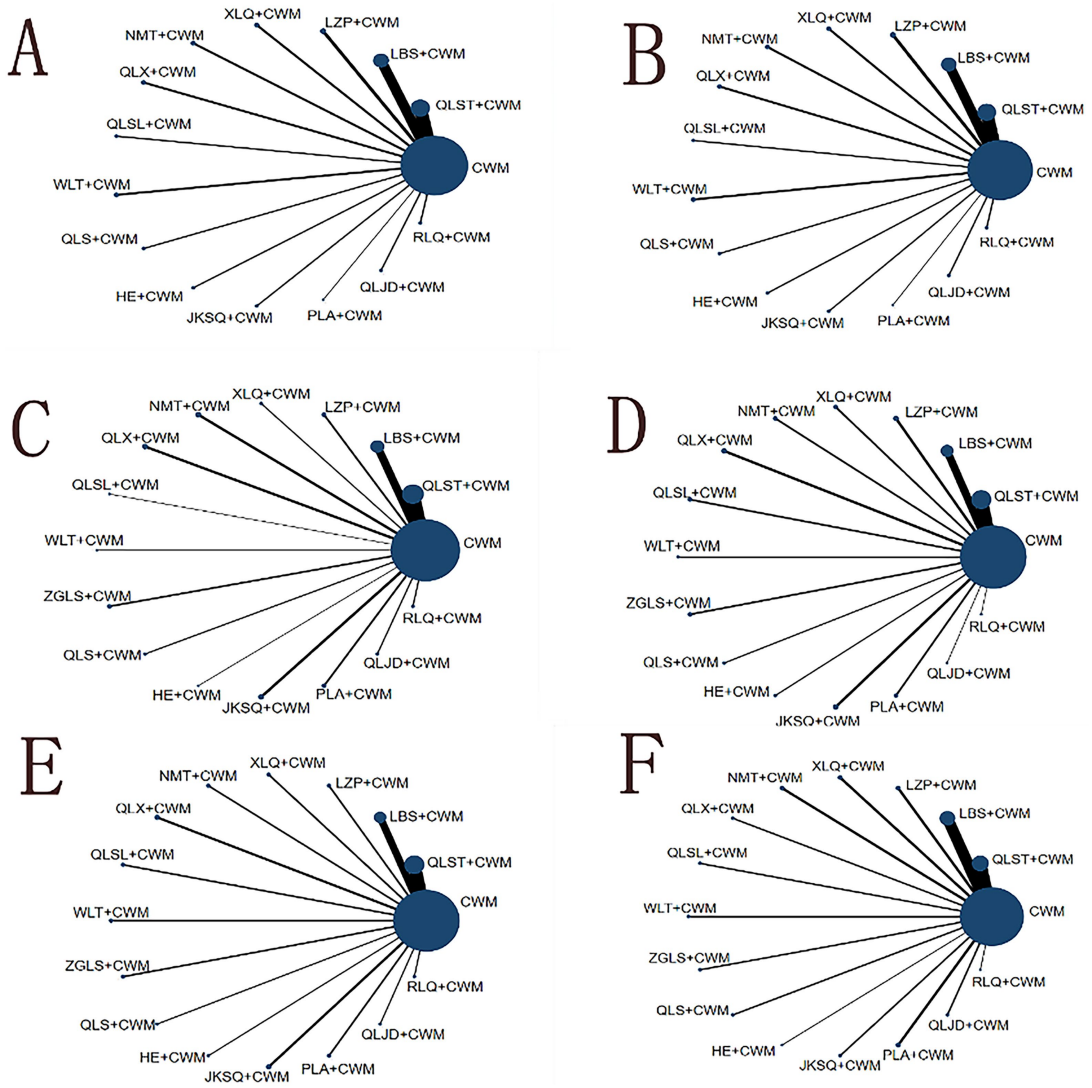
Network graph of outcomes. The blue nodes represent the total number of treatments. The line thickness corresponds to the number of comparison trials. **(A)** Total efficiency ratio; **(B)** prostate volume; **(C)** residure volume; **(D)** maximum urinary flow rate; **(E)** international prostate symptom score; **(F)** adverse reactions. CWM, conventional western medicine; QLST, Qianliesutong capsule; LBS, Longbisu capsule; LZP, Lingzepian tablet; XLQ, Xialiqi capsule; NMT, Ningmitai capsule; QLX, Qianliexin capsule; QLSL, Qianliesule capsule; WLT, Wenglitong capsule; ZGLS, Zeguilongsu capsule; QLS, Qianliesu capsule; HE, Huange capsule; JKSQ, Jinkuishenqi capsule; PLA, Puleai capsule, QLJD, Qianliejindan capsule; RLQ, Relinqing granules.

All 56 pieces of research including 14 OCPMs and 14 interventions recorded the total efficiency ratio of BPH (Figure 4A).

Compared with CWM alone, QLST + CWM (MD 3.95, 95% CI 2.74–5.70, low certainty), LBS + CWM (MD 2.87, 95% CI 2.06–3.99, very low certainty), LZP + CWM (MD 2.63, 95% CI 1.56–4.40, low certainty), XLQ + CWM (MD 5.33, 95% CI 2.20–12.92, very low certainty), NMT + CWM (MD 5.14, 95% CI 1.96–13.48, low certainty), QLX + CWM (MD 4.45, 95% CI 1.91–10.40, very low certainty), QLSL + CWM (MD 3.02, 95% CI 1.32–6.89, low certainty), WLT+ CWM (MD 2.67, 95% CI 1.31–5.41, very low certainty), QLS + CWM (MD 4.57, 95% CI 1.38–15.39, very low certainty), HE+ CWM (MD 3.76, 95% CI 1.74–8.14, low certainty), JKSQ+ CWM (MD 6.04, 95% CI 1.86–19.61, low certainty), PLA + CWM (MD 4.56, 95% CI 1.12–18.46, very low certainty), RLQ + CWM (MD 3.45, 95% CI 1.55–7.68, very low certainty) were beneficial to rise total efficiency ratio (Table 2). Compared with CWM alone, there was no significant difference in RLQ + CWM in the treatment of BPH (Table 2).

**Table 2:** Results of the network meta-analysis of the total efficiency ratio.

														
CWM														
3.95 (2.74,5.70)	QLST+CWM													
2.87 (2.06,3.99)	0.73 (0.44,1.19)	LBS + CWM												
2.63 (1.56,4.40)	0.66 (0.35,1.25)	0.92 (0.51,1.65)	LZP + CWM											
5.33 (2.20,12.92)	1.35 (0.52,3.52)	1.86 (0.72,4.78)	2.03 (0.73,5.66)	XLQ + CWM										
5.14 (1.96,13.48)	1.30 (0.46,3.65)	1.79 (0.65,4.96)	1.96 (0.66,5.85)	0.96 (0.26,3.57)	NMT + CWM									
4.45 (1.91,10.40)	1.13 (0.45,2.84)	1.55 (0.63,3.85)	1.70 (0.63,4.57)	0.84 (0.25,2.84)	0.87 (0.24,3.13)	QLX + CWM								
3.02 (1.32,6.89)	0.76 (0.31,1.89)	1.05 (0.43,2.56)	1.15 (0.43,3.05)	0.57 (0.17,1.90)	0.59 (0.16,2.09)	0.68 (0.21,2.21)	QLSL+CWM							
2.67 (1.31,5.41)	0.68 (0.30,1.50)	0.93 (0.43,2.02)	1.02 (0.42,2.43)	0.50 (0.16,1.55)	0.52 (0.16,1.71)	0.60 (0.20,1.80)	0.88 (0.30,2.62)	WLT + CWM						
4.57 (1.38,15.09)	1.16 (0.33,4.04)	1.59 (0.46,5.50)	1.74 (0.47,6.39)	0.86 (0.19,3.79)	0.89 (0.19,4.13)	1.03 (0.24,4.44)	1.51 (0.35,6.47)	1.71 (0.43,6.87)	QLS + CWM					
3.76 (1.74,8.14)	0.95 (0.40,2.24)	1.31 (0.57,3.04)	1.43 (0.56,3.63)	0.70 (0.22,2.28)	0.73 (0.21,2.52)	0.84 (0.27,2.66)	1.25 (0.40,3.86)	1.41 (0.49,4.02)	0.82 (0.20,3.41)	HE+CWM				
6.04 (1.86,19.61)	1.53 (0.45,5.25)	2.11 (0.62,7.15)	2.30 (0.64,8.31)	1.13 (0.26,4.94)	1.18 (0.26,5.38)	1.36 (0.32,5.78)	2.00 (0.48,8.44)	2.27 (0.57,8.94)	1.32 (0.25,7.07)	1.61 (0.39,6.57)	JKSQ+CWM			
4.56 (1.12,18.46)	1.15 (0.27,4.90)	1.59 (0.38,6.69)	1.74 (0.39,7.71)	0.85 (0.16,4.47)	0.89 (0.16,4.85)	1.02 (0.20,5.25)	1.51 (0.30,7.66)	1.71 (0.36,8.19)	1.00 (0.16,6.27)	1.21 (0.25,5.99)	0.75 (0.12,4.69)	PLA + CWM		
2.50 (0.98,6.36)	0.63 (0.23,1.73)	0.87 (0.32,2.35)	0.95 (0.33,2.78)	0.47 (0.13,1.70)	0.49 (0.13,1.86)	0.56 (0.16,1.98)	0.83 (0.24,2.88)	0.94 (0.29,3.03)	0.55 (0.12,2.49)	0.66 (0.20,2.23)	0.41 (0.09,1.86)	0.55 (0.10,2.95)	QLJD+CWM	
3.45 (1.55,7.68)	0.87 (0.36,2.11)	1.20 (0.50,2.87)	1.31 (0.51,3.42)	0.65 (0.20,2.13)	0.67 (0.19,2.35)	0.77 (0.24,2.49)	1.14 (0.36,3.61)	1.29 (0.44,3.77)	0.75 (0.18,3.18)	0.92 (0.30,2.79)	0.57 (0.14,2.37)	0.76 (0.15,3.79)	1.38 (0.40,4.72)	RLQ + CWM

Brown indicates the drug. Blue color indicates the corresponding value.

**Figure 5:**
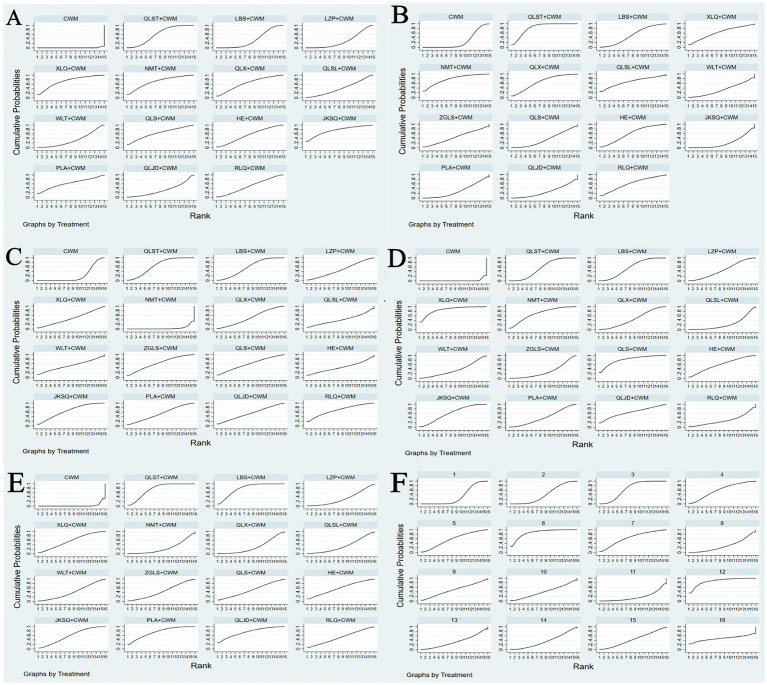
Plots of the surface under the cumulative ranking curves for all treatments **(A)** Total efficiency ratio; **(B)** prostate volume; **(C)** residure volume; **(D)** maximum urinary flow rate; **(E)** international prostate symptom score; **(F)** adverse reactions.

Based on the SUCRA values, JKSQ + CWM had the highest probability for rise total efficiency ratio (SUCRA: 76.5%), followed by XLQ + CWM (SUCRA: 74.2%), and NMT + CWM (SUCRA: 71.6%; Figure 5A; Table 3).

**Table 3:** Surface under the cumulative ranking curve and ranking probability of different Chinese patent medicines on each outcome.

Treatment	efficacy		PV		PVR		Qmax		IPSS		safety	
	SUCRA(%)	Rank	SUCRA(%)	Rank	SUCRA(%)	Rank	SUCRA(%)	Rank	SUCRA(%)	Rank	SUCRA(%)	Rank
CWM	0.5	15	14.7	14	21.4	15	1.6	16	2.8	16	32.7	14
QLST+CWM	59.9	7	61.2	5	65.7	2	54.9	8	75.8	4	41.8	10
LBS + CWM	35.1	11	32	13	59.1	6	57.9	7	79.7	1	65.9	3
LZP + CWM	30.7	14	–	–	48	11	47.3	9	33.5	13	61	5
XLQ + CWM	74.2	2	44.3	11	47.1	12	87.2	1	56.2	7	58.1	6
NMT + CWM	71.6	3	59.4	6	3.7	16	71.1	3	25	15	87	2
QLX + CWM	64	4	45.5	10	50.3	10	46	10	31.5	14	64.1	4
QLSL+CWM	41.9	10	51.7	8	41.4	14	24.3	15	34.2	12	29.4	15
WLT + CWM	33.6	12	0.7	15	52.7	9	37.4	12	42.6	11	49.1	7
ZGLS+CWM	–	–	58.3	7	63.6	4	31.4	13	44.6	10	41	11
QLS + CWM	64	4	68.1	3	59.5	5	81.8	2	50.1	9	18.2	16
HE+CWM	54.8	8	87.7	1	44.8	13	59.6	6	59.4	6	87.2	1
JKSQ+CWM	76.5	1	48.6	9	65.7	2	61.1	5	59.8	5	35	13
PLA + CWM	61.9	6	63	4	53.2	8	42.4	11	76.4	3	36.5	12
QLJD+CWM	31.7	13	33.6	12	54.7	7	65.6	4	76.5	2	44.4	9
RLQ + CWM	49.6	9	81.2	2	68.9	1	30.3	14	51.9	8	48.7	8

Red indicates the top 3. Green is the drug corresponding to the red anti-variation distinction.

#### 3.4.2 Prostate volume

All 40 pieces of research including 14 OCPMs and 14 interventions recorded the prostate volume of BPH (Figure 4B).

Compared with CWM alone, QLST + CWM (MD −5.01, 95% CI −6.42–−3.59, low certainty), NMT + CWM (MD −5.03, 95% CI −8.73–−1.32, low certainty), QLX + CWM (MD −3.65, 95% CI −6.43–−0.86, very low certainty), QLS + CWM (MD −6.12, 95% CI −9.78–−2.46, very low certainty), HE + CWM (MD −9.18, 95% CI −12.74–−5.63, low certainty), JKSQ + CWM (MD −3.90, 95% CI −7.08–−0.73, low certainty), PLA + CWM (MD −5.42, 95% CI −9.66–−1.19, very low certainty), RLQ + CWM (MD −8.91, 95% CI −14.73–−3.09, very low certainty) were beneficial to reduce prostate volume (Table 4). Compared with CWM alone, there were no significant differences between LBS + CWM, XLQ + CWM, QLSL + CWM, WLT + CWM, ZGLS + CWM, and QLJD + CWM in the treatment of BPH (Table 4).

**Table 4:** Results of the network meta-analysis of the prostate volume.

														
CWM														
−5.01 (−6.42,−3.59)	QLST+CWM													
−2.10 (−4.29,0.09)	2.90 (0.30,5.51)	LBS + CWM												
−3.42 (−7.76,0.92)	1.59 (−2.98,6.15)	−1.32 (−6.18,3.54)	XLQ + CWM											
−5.03 (−8.73,−1.32)	−0.02 (−3.98,3.94)	−2.92 (−7.23,1.38)	−1.61 (−7.31,4.10)	NMT + CWM										
−3.65 (−6.43,−0.86)	1.36 (−1.76,4.48)	−1.54 (−5.08,2.00)	−0.23 (−5.38,4.93)	1.38 (−3.25,6.01)	QLX + CWM									
−4.12 (−9.32,1.08)	0.89 (−4.50,6.27)	−2.02 (−7.66,3.62)	−0.70 (−7.47,6.07)	0.91 (−5.47,7.29)	−0.47 (−6.37,5.42)	QLSL+CWM								
−2.43 (−6.04,1.18)	2.58 (−1.30,6.45)	−0.33 (−4.54,3.89)	0.99 (−4.65,6.63)	2.60 (−2.57,7.77)	1.22 (−3.33,5.77)	1.69 (−4.63,8.02)	WLT + CWM							
−5.00 (−10.36,0.36)	0.01 (−5.54,5.55)	−2.90 (−8.69,2.90)	−1.58 (−8.48,5.32)	0.03 (−6.49,6.54)	−1.35 (−7.40,4.69)	−0.88 (−8.35,6.59)	−2.57 (−9.03,3.89)	ZGLS+CWM						
−6.12 (−9.78,−2.46)	−1.11 (−5.03,2.81)	−4.01 (−8.28,0.25)	−2.70 (−8.37,2.98)	−1.09 (−6.30,4.12)	−2.47 (−7.07,2.13)	−2.00 (−8.35,4.36)	−3.69 (−8.83,1.45)	−1.12 (−7.61,5.38)	QLS + CWM					
−9.18 (−12.74,−5.63)	−4.18 (−8.01,−0.35)	−7.08 (−11.26,−2.90)	−5.76 (−11.38,−0.15)	−4.16 (−9.29,0.98)	−5.54 (−10.06,−1.02)	−5.06 (−11.36,1.23)	−6.75 (−11.82,−1.69)	−4.18 (−10.62,2.25)	−3.07 (−8.17,2.04)	HE+CWM				
−3.90 (−7.08,−0.73)	1.10 (−2.37,4.58)	−1.80 (−5.66,2.06)	−0.48 (−5.86,4.89)	1.12 (−3.76,6.00)	−0.26 (−4.48,3.96)	0.22 (−5.87,6.31)	−1.47 (−6.28,3.33)	1.10 (−5.14,7.33)	2.21 (−2.63,7.06)	5.28 (0.51,10.05)	JKSQ+CWM			
−5.42 (−9.66,−1.19)	−0.42 (−4.89,4.05)	−3.32 (−8.09,1.45)	−2.00 (−8.07,4.06)	−0.40 (−6.02,5.23)	−1.78 (−6.85,3.30)	−1.30 (−8.01,5.40)	−2.99 (−8.56,2.57)	−0.42 (−7.26,6.41)	0.69 (−4.91,6.29)	3.76 (−1.77,9.29)	−1.52 (−6.82,3.78)	PLA + CWM		
−1.66 (−5.55,2.22)	3.34 (−0.80,7.48)	0.44 (−4.03,4.90)	1.75 (−4.08,7.59)	3.36 (−2.01,8.73)	1.98 (−2.81,6.77)	2.46 (−4.03,8.95)	0.76 (−4.54,6.07)	3.34 (−3.29,9.96)	4.45 (−0.89,9.79)	7.52 (2.25,12.79)	2.24 (−2.78,7.26)	3.76 (−1.99,9.51)	QLJD+CWM	
−8.91 (−14.73,−3.09)	−3.90 (−9.90,2.09)	−6.81 (−13.03,−0.59)	−5.49 (−12.75,1.77)	−3.88 (−10.78,3.02)	−5.26 (−11.72,1.19)	−4.79 (−12.59,3.01)	−6.48 (−13.33,0.37)	−3.91 (−11.82,4.00)	−2.79 (−9.67,4.08)	0.27 (−6.55,7.10)	−5.01 (−11.64,1.63)	−3.49 (−10.69,3.71)	−7.25 (−14.25,−0.25)	RLQ + CWM

Brown indicates the drug. Blue color indicates the corresponding value.

Based on the SUCRA values, HE + CWM had the highest probability of reducing prostate volume. (SUCRA: 87.7%), followed by RLQ + CWM (SUCRA: 81.2%), and QLS + CWM (SUCRA: 68.1%; Figure 5B; Table 3).

#### 3.4.3 Residure volume

All 61 pieces of research including 15 OCPMs and 15 interventions recorded the residure volume of BPH (Figure 4C).

Compared with CWM alone, QLST + CWM (MD −8.90, 95% CI −13.76–−4.05, low certainty), LBS + CWM (MD −7.72, 95% CI −14.00–−1.44, very low certainty) were beneficial to reduce residure volume (Table 5). Compared with CWM alone, there were no significant differences in others in the treatment of BPH (Table 5).

**Table 5:** Results of the network meta-analysis of the residure volume.

															
CWM															
−8.90 (−13.76,−4.05)	QLST+CWM														
−7.72 (−14.00,−1.44)	1.18 (−6.76,9.12)	LBS + CWM													
−5.22 (−16.96,6.53)	3.69 (−9.02,16.40)	2.50 (−10.82,15.82)	LZP + CWM												
−4.76 (−19.40,9.87)	4.14 (−11.28,19.56)	2.96 (−12.97,18.88)	0.46 (−18.31,19.22)	XLQ + CWM											
10.31 (−0.30,20.92)	19.21 (7.55,30.88)	18.03 (5.70,30.36)	15.53 (−0.30,31.36)	15.07 (−3.00,33.15)	NMT + CWM										
−6.05 (−16.35,4.24)	2.85 (−8.53,14.23)	1.67 (−10.39,13.73)	−0.84 (−16.45,14.78)	−1.29 (−19.18,16.60)	−16.36 (−31.15,−1.58)	QLX + CWM									
−2.64 (−23.00,17.72)	6.26 (−14.67,27.20)	5.08 (−16.23,26.39)	2.58 (−20.93,26.09)	2.12 (−22.95,27.20)	−12.95 (−35.91,10.01)	3.41 (−19.40,26.23)	QLSL+CWM								
−6.46 (−26.81,13.89)	2.44 (−18.48,23.37)	1.26 (−20.04,22.56)	−1.24 (−24.74,22.26)	−1.70 (−26.77,23.37)	−16.77 (−39.73,6.18)	−0.41 (−23.22,22.40)	−3.82 (−32.61,24.97)	WLT + CWM							
−8.72 (−20.57,3.13)	0.19 (−12.62,12.99)	−1.00 (−14.41,12.42)	−3.50 (−20.19,13.19)	−3.96 (−22.79,14.88)	−19.03 (−34.94,−3.12)	−2.66 (−18.36,13.03)	−6.08 (−29.64,17.48)	−2.26 (−25.81,21.30)	ZGLS+CWM						
−8.21 (−22.65,6.23)	0.69 (−14.54,15.92)	−0.49 (−16.23,15.25)	−2.99 (−21.60,15.62)	−3.45 (−24.00,17.11)	−18.52 (−36.44,−0.60)	−2.16 (−19.89,15.57)	−5.57 (−30.53,19.39)	−1.75 (−26.70,23.20)	0.51 (−18.17,19.18)	QLS + CWM					
−4.29 (−24.65,16.07)	4.61 (−16.31,25.54)	3.43 (−17.87,24.73)	0.93 (−22.57,24.43)	0.47 (−24.60,25.54)	−14.60 (−37.56,8.36)	1.76 (−21.05,24.57)	−1.65 (−30.44,27.14)	2.17 (−26.62,30.96)	4.43 (−19.13,27.98)	3.92 (−21.04,28.87)	HE+CWM				
−9.33 (−19.56,0.90)	−0.42 (−11.75,10.90)	−1.61 (−13.62,10.40)	−4.11 (−19.69,11.47)	−4.57 (−22.42,13.29)	−19.64 (−34.39,−4.89)	−3.28 (−17.79,11.24)	−6.69 (−29.48,16.10)	−2.87 (−25.65,19.91)	−0.61 (−16.27,15.05)	−1.12 (−18.81,16.58)	−5.04 (−27.82,17.74)	JKSQ+CWM			
−6.63 (−18.59,5.32)	2.27 (−10.63,15.17)	1.09 (−12.42,14.59)	−1.42 (−18.17,15.34)	−1.87 (−20.77,17.02)	−16.94 (−32.93,−0.96)	−0.58 (−16.35,15.19)	−3.99 (−27.61,19.62)	−0.17 (−23.78,23.43)	2.08 (−14.75,18.91)	1.58 (−17.17,20.32)	−2.34 (−25.95,21.26)	2.69 (−13.04,18.43)	PLA + CWM		
−6.95 (−21.38,7.48)	1.95 (−13.27,17.18)	0.77 (−14.97,16.51)	−1.73 (−20.34,16.87)	−2.19 (−22.74,18.36)	−17.26 (−35.17,0.65)	−0.90 (−18.62,16.83)	−4.31 (−29.27,20.65)	−0.49 (−25.44,24.46)	1.77 (−16.91,20.44)	1.26 (−19.15,21.67)	−2.66 (−27.61,22.29)	2.38 (−15.31,20.07)	−0.32 (−19.05,18.42)	QLJD+CWM	
−10.65 (−25.56,4.26)	−1.75 (−17.42,13.93)	−2.93 (−19.11,13.25)	−5.43 (−24.41,13.54)	−5.89 (−26.78,15.00)	−20.96 (−39.25,−2.67)	−4.60 (−22.71,13.52)	−8.01 (−33.25,17.22)	−4.19 (−29.42,21.04)	−1.93 (−20.98,17.11)	−2.44 (−23.19,18.31)	−6.36 (−31.59,18.87)	−1.32 (−19.40,16.76)	−4.02 (−23.12,15.09)	−3.70 (−24.45,17.05)	RLQ + CWM

Brown indicates the drug. Blue color indicates the corresponding value.

Based on the SUCRA values, QLST + CWM had the highest probability of reducing residure volume (SUCRA: 65.7%), followed by LBS+ CWM (SUCRA: 59.1%; Figure 5C; Table 3).

#### 3.4.4 Maximum urinary flow rate

All 64 pieces of research including 15 OCPMs and 15 interventions recorded the maximum urinary flow rate of BPH (Figure 4D).

Compared with CWM alone, QLST + CWM (MD 3.27, 95% CI 2.46–4.08, low certainty), LBS + CWM (MD 3.38, 95% CI 2.30–4.45, very low certainty), LZP + CWM (MD 2.98, 95% CI 1.26–4.70, low certainty), XLQ + CWM (MD 5.00, 95% CI 2.78–7.23, very low certainty), NMT + CWM (MD 3.99, 95% CI 1.98–5.99, low certainty), QLX + CWM (MD 2.90, 95% CI 1.18–4.61, very low certainty), ZGLS+ CWM (MD 2.21, 95% CI 0.16–4.26, very low certainty), QLS + CWM (MD 4.67, 95% CI 2.26–7.08, very low certainty), HE + CWM (MD 3.51, 95% CI 1.05–5.97, low certainty), JKSQ+ CWM (MD 3.52, 95% CI 1.83–5.22, low certainty), PLA + CWM (MD 2.76, 95% CI 0.70–4.82, very low certainty), QLJD + CWM (MD 3.94, 95% CI 0.32–7.56, very low certainty) were beneficial to rise maximum urinary flow rate (Table 6). Compared with CWM alone, there were no significant differences between QLSL + CWM, WLT + CWM, RLQ + CWM in the treatment of BPH (Table 6).

**Table 6:** Results of the network meta-analysis of the maximum urinary flow rate.

															
CWM															
3.27 (2.46,4.08)	QLST+CWM														
3.38 (2.30,4.45)	0.11 (−1.24,1.45)	LBS + CWM													
2.98 (1.26,4.70)	−0.29 (−2.19,1.61)	−0.40 (−2.43,1.63)	LZP + CWM												
5.00 (2.78,7.23)	1.74 (−0.63,4.10)	1.63 (−0.85,4.10)	−2.02 (−4.84,0.79)	XLQ + CWM											
3.99 (1.98,5.99)	0.72 (−1.44,2.88)	0.61 (−1.66,2.88)	−1.01 (−3.65,1.63)	−1.02 (−4.01,1.98)	NMT + CWM										
2.90 (1.18,4.61)	−0.37 (−2.27,1.53)	−0.48 (−2.50,1.55)	0.08 (−2.35,2.51)	−2.11 (−4.92,0.71)	−1.09 (−3.73,1.55)	QLX + CWM									
1.84 (−0.29,3.97)	−1.43 (−3.70,0.85)	−1.54 (−3.92,0.85)	1.14 (−1.60,3.88)	−3.16 (−6.24,−0.08)	−2.15 (−5.07,0.78)	−1.06 (−3.79,1.68)	QLSL+CWM								
2.44 (−0.03,4.91)	−0.83 (−3.43,1.78)	−0.94 (−3.63,1.76)	0.54 (−2.47,3.55)	−2.56 (−5.89,0.76)	−1.55 (−4.73,1.64)	−0.46 (−3.47,2.55)	0.60 (−2.66,3.86)	WLT + CWM							
2.21 (0.16,4.26)	−1.06 (−3.26,1.14)	−1.17 (−3.48,1.14)	0.77 (−1.90,3.45)	−2.80 (−5.82,0.23)	−1.78 (−4.65,1.08)	−0.69 (−3.36,1.98)	0.37 (−2.59,3.32)	−0.23 (−3.45,2.98)	ZGLS+CWM						
4.67 (2.26,7.08)	1.41 (−1.13,3.95)	1.30 (−1.34,3.93)	−1.69 (−4.65,1.27)	−0.33 (−3.61,2.95)	0.69 (−2.45,3.82)	1.78 (−1.18,4.73)	2.83 (−0.38,6.05)	2.23 (−1.22,5.69)	2.47 (−0.70,5.63)	QLS + CWM					
3.51 (1.05,5.97)	0.25 (−2.34,2.83)	0.14 (−2.55,2.82)	−0.53 (−3.53,2.47)	−1.49 (−4.80,1.82)	−0.47 (−3.65,2.70)	0.61 (−2.38,3.61)	1.67 (−1.58,4.93)	1.07 (−2.42,4.56)	1.31 (−1.89,4.51)	−1.16 (−4.60,2.28)	HE+CWM				
3.52 (1.83,5.22)	0.25 (−1.62,2.13)	0.14 (−1.86,2.15)	−0.54 (−2.96,1.87)	−1.48 (−4.28,1.31)	−0.47 (−3.09,2.16)	0.62 (−1.79,3.03)	1.68 (−1.04,4.40)	1.08 (−1.92,4.08)	1.31 (−1.34,3.97)	−1.15 (−4.10,1.79)	0.01 (−2.98,2.99)	JKSQ+CWM			
2.76 (0.70,4.82)	−0.51 (−2.73,1.70)	−0.62 (−2.95,1.70)	0.22 (−2.46,2.91)	−2.25 (−5.28,0.78)	−1.23 (−4.11,1.64)	−0.14 (−2.82,2.54)	0.91 (−2.05,3.88)	0.32 (−2.90,3.53)	0.55 (−2.36,3.45)	−1.92 (−5.09,1.25)	−0.76 (−3.96,2.45)	−0.77 (−3.43,1.90)	PLA + CWM		
3.94 (0.32,7.56)	0.67 (−3.04,4.39)	0.56 (−3.22,4.34)	−0.96 (−4.97,3.05)	−1.06 (−5.32,3.19)	−0.05 (−4.19,4.09)	1.04 (−2.97,5.05)	2.10 (−2.10,6.30)	1.50 (−2.89,5.89)	1.73 (−2.43,5.90)	−0.73 (−5.09,3.62)	0.43 (−3.95,4.81)	0.42 (−3.58,4.42)	1.18 (−2.98,5.35)	QLJD+CWM	
1.93 (−1.47,5.33)	−1.34 (−4.83,2.16)	−1.45 (−5.01,2.12)	1.05 (−2.76,4.86)	−3.07 (−7.14,0.99)	−2.06 (−6.00,1.89)	−0.97 (−4.78,2.84)	0.09 (−3.92,4.10)	−0.51 (−4.71,3.69)	−0.28 (−4.25,3.69)	−2.74 (−6.91,1.42)	−1.58 (−5.78,2.61)	−1.59 (−5.39,2.21)	−0.83 (−4.80,3.15)	−2.01 (−6.98,2.96)	RLQ + CWM

Brown indicates the drug. Blue color indicates the corresponding value.

Based on the SUCRA values, XLQ + CWM had the highest probability of elevating the maximum urinary flow rate (SUCRA: 87.2%), followed by QLS + CWM (SUCRA: 81.8%), and NMT + CWM (SUCRA: 71.1%; Figure 5D; Table 3).

#### 3.4.5 IPSS

All 67 pieces of research including 15 OCPMs and 15 interventions recorded the IPSS of BPH (Figure 4E).

Compared with CWM alone, QLST + CWM (MD −4.84, 95% CI −6.03–−3.65, low certainty), LBS + CWM (MD −5.09, 95% CI −6.66–−3.51, very low certainty), XLQ + CWM (MD −3.86, 95% CI −6.87–−0.85, very low certainty), ZGLS + CWM (MD −3.08, 95% CI −6.05–−0.03, very low certainty), HE+ CWM (MD −4.08, 95% CI −7.74–−0.43 low certainty), JKSQ + CWM (MD −3.98, 95% CI −6.55–−1.41, low certainty), PLA + CWM (MD −5.11, 95% CI −8.16–−2.05, very low certainty), QLJD+ CWM (MD −5.23, 95% CI −9.03–−1.42, very low certainty) were beneficial to reduce IPSS (Table 7). Compared with CWM alone, there were no significant differences between LZP + CWM, NMT + CWM, QLX + CWM, QLSL+ CWM, QLS + CWM,WLT + CWM, and RLQ + CWM in the treatment of BPH (Table 7).

**Table 7:** Results of the network meta-analysis of the international prostate symptom score.

															
CWM															
−4.84 (−6.03,−3.65)	QLST+CWM														
−5.09 (−6.66,−3.51)	−0.25 (−2.22,1.73)	LBS + CWM													
−2.39 (−5.35,0.57)	2.45 (−0.74,5.64)	2.70 (−0.65,6.05)	LZP + CWM												
−3.86 (−6.87,−0.85)	0.98 (−2.26,4.21)	1.23 (−2.17,4.62)	−1.47 (−5.69,2.75)	XLQ + CWM											
−1.79 (−4.77,1.19)	3.05 (−0.16,6.26)	3.30 (−0.07,6.67)	0.60 (−3.60,4.80)	2.07 (−2.17,6.30)	NMT + CWM										
−2.29 (−4.86,0.28)	2.55 (−0.28,5.39)	2.80 (−0.21,5.82)	0.10 (−3.81,4.02)	1.57 (−2.38,5.53)	−0.49 (−4.43,3.44)	QLX + CWM									
−2.42 (−5.47,0.64)	2.43 (−0.85,5.71)	2.67 (−0.77,6.11)	−0.02 (−4.28,4.23)	1.45 (−2.84,5.74)	−0.62 (−4.89,3.65)	−0.13 (−4.12,3.87)	QLSL+CWM								
−2.97 (−6.05,0.11)	1.87 (−1.43,5.17)	2.12 (−1.34,5.58)	−0.58 (−4.85,3.69)	0.89 (−3.41,5.20)	−1.18 (−5.46,3.11)	−0.68 (−4.69,3.33)	−0.55 (−4.89,3.78)	WLT + CWM							
−3.08 (−6.13,−0.03)	1.76 (−1.51,5.03)	2.01 (−1.42,5.44)	−0.69 (−4.93,3.56)	0.78 (−3.50,5.07)	−1.29 (−5.55,2.98)	−0.79 (−4.78,3.20)	−0.66 (−4.98,3.65)	−0.11 (−4.44,4.22)	ZGLS+CWM						
−3.47 (−7.07,0.14)	1.37 (−2.42,5.17)	1.62 (−2.31,5.55)	−1.08 (−5.74,3.59)	0.39 (−4.30,5.09)	−1.67 (−6.35,3.00)	−1.18 (−5.61,3.25)	−1.05 (−5.78,3.67)	−0.50 (−5.24,4.24)	−0.39 (−5.11,4.33)	QLS + CWM					
−4.08 (−7.74,−0.43)	0.76 (−3.08,4.60)	1.01 (−2.97,4.98)	−1.69 (−6.39,3.01)	−0.22 (−4.95,4.51)	−2.29 (−7.00,2.43)	−1.79 (−6.26,2.67)	−1.67 (−6.43,3.10)	−1.11 (−5.89,3.66)	−1.00 (−5.76,3.75)	−0.61 (−5.75,4.52)	HE+CWM				
−3.98 (−6.55,−1.41)	0.86 (−1.97,3.69)	1.11 (−1.91,4.12)	−1.59 (−5.51,2.33)	−0.12 (−4.08,3.84)	−2.19 (−6.12,1.75)	−1.69 (−5.33,1.94)	−1.57 (−5.56,2.43)	−1.01 (−5.02,3.00)	−0.90 (−4.89,3.08)	−0.51 (−4.94,3.91)	0.10 (−4.37,4.57)	JKSQ+CWM			
−5.11 (−8.16,−2.05)	−0.27 (−3.55,3.01)	−0.02 (−3.46,3.42)	−2.72 (−6.97,1.53)	−1.25 (−5.53,3.04)	−3.32 (−7.58,0.95)	−2.82 (−6.81,1.17)	−2.69 (−7.02,1.63)	−2.14 (−6.48,2.20)	−2.03 (−6.35,2.29)	−1.64 (−6.37,3.08)	−1.03 (−5.79,3.74)	−1.13 (−5.12,2.86)	PLA + CWM		
−5.23 (−9.03,−1.42)	−0.39 (−4.37,3.60)	−0.14 (−4.26,3.98)	−2.84 (−7.66,1.98)	−1.37 (−6.22,3.49)	−3.43 (−8.27,1.40)	−2.94 (−7.53,1.65)	−2.81 (−7.69,2.07)	−2.26 (−7.15,2.64)	−2.15 (−7.02,2.73)	−1.76 (−7.00,3.48)	−1.15 (−6.42,4.13)	−1.25 (−5.84,3.35)	−0.12 (−5.00,4.76)	QLJD+CWM	
−3.56 (−7.25,0.13)	1.28 (−2.59,5.16)	1.53 (−2.48,5.54)	−1.17 (−5.90,3.56)	0.30 (−4.46,5.06)	−1.77 (−6.51,2.98)	−1.27 (−5.77,3.22)	−1.14 (−5.93,3.65)	−0.59 (−5.39,4.21)	−0.48 (−5.27,4.30)	−0.09 (−5.25,5.07)	0.52 (−4.67,5.71)	0.42 (−4.07,4.92)	1.55 (−3.24,6.34)	1.67 (−3.63,6.97)	RLQ + CWM

Brown indicates the drug. Blue color indicates the corresponding value.

Based on the SUCRA values, LBS + CWM had the highest probability of reducing IPSS (SUCRA: 79.7%), followed by QLJD + CWM (SUCRA: 76.5%), and PLA+ CWM (SUCRA: 76.4%; Figure 5E; Table 3).

#### 3.4.6 Adverse reactions

Of the 72 included studies, 53 reported the occurrence of adverse reactions. Ten studies reported no adverse reactions in both experimental and control groups, while the remaining 43 studies reported example adverse events. Adverse reactions included gastrointestinal reactions such as nausea, vomiting, diarrhea, gastrointestinal discomfort, dizziness, hypotension, rash, impotence, as well as mild hepatic and renal impairment. (Figure 4F; Table 8).

**Table 8:** Occurrence of adverse reactions.

Study ID	Adverse reactions		Intervention		Response
	Experiment	Control	Experiment	Control	
Gao et al. (2022) (11)	abdominal pain (1 case), nauseating (2 cases)	nauseating (1 case), spin(1 case)	QLST+CWM	CWM	The experimental drug was taken 30 min after the meal, and the symptoms were relieve.
Li et al. (2020) (12)	0	0	QLST+CWM	CWM	
Tong et al. (2020) (13)	diarrhoe (1 case), vomiting (2 cases)	diarrhoe (3 cases)、vomiting(4 cases), spin(1 cases)	QLST+CWM	CWM	
Kong et al. (2019) (14)	nauseating (3 cases), vomiting (4 cases)	nauseating (2 cases), vomiting(1 cases)	QLST+CWM	CWM	
Liang et al. (2018) (17)	diarrhoe (1 case), impotence (1 case), painful urination (1 cases)	diarrhoe (2 cases), impotence(1 case), painful urination (1 case)	QLST+CWM	CWM	
Tian et al. (2018) (18)	0	0	QLST+CWM	CWM	
Wan (2017) (19)	spin (4 cases), nauseating (1 case)	spin (4 cases), nauseating(3 cases)	QLST+CWM	CWM	
Man et al. (2017) (21)	0	0	QLST+CWM	CWM	
Shi et al. (2016) (23)	0	0	QLST+CWM	CWM	
Li et al. (2015) (24)	spin (5 cases), nauseating (3 cases)	spin (3 cases), nauseating(3 cases)	QLST+CWM	CWM	
Fu et al. (2015) (25)	nauseating	nauseating	QLST+CWM	CWM	
Xuan et al. (2012) (26)	spin (2 cases), hypotension (1 case)	spin (2cases), nauseating(2 cases)	QLST+CWM	CWM	
Ma et al. (2009) (27)	spin (15 cases)	spin (16 cases)	QLST+CWM	CWM	
Zhang et al. (2022) (29)	spin (1 case), nauseating (2 cases)	spin (2 cases), nauseating(1 case)	LBS + CWM	CWM	
Xu et al. (2021) (30)	hypotension (2 cases), nauseating (2 cases)	hypotension (3 cases), nauseating (1 case)	LBS + CWM	CWM	
Lu et al. (2020) (31)	hypotension (1 cases), nauseating (1 case)	spin (4 cases), nauseating(3 cases), others (32 cases)	LBS + CWM	CWM	
Zhou et al. (2018) (32)	hypotension (1 case), nauseating (1 case), insomnia (1 case)	headaches (2 cases), nauseating(1 case)	LBS + CWM	CWM	
Ji et al. (2018) (33)	nauseating (15 cases), abdominal pain (10 cases)	nauseating (19 cases), abdominal pain(13 cases)	LBS + CWM	CWM	
Chen et al. (2017) (34)	diarrhoe (1 case), hypotension (1 case), nauseating (1 case)	diarrhoe (1 case), hypotension (2 case nauseating), nauseating (1 case)	LBS + CWM	CWM	
Yuan (2017) (35)	nauseating (4 cases), spin(1 case), hypotension(2 case)	nauseating (3 cases), spin(2 cases), hypotension (1 case)	LBS + CWM	CWM	
Xue et al. (2017) (37)	drowsiness (1 case)	spin (2 cases)	LBS + CWM	CWM	
Song et al. (2016) (38)	3 cases	8 cases	LBS + CWM	CWM	
Zhang (2016) (39)	nauseating (1 case)	nauseating (2 cases)	LBS + CWM	CWM	
Chang et al. (2015) (40)	spin (2 cases), abdominal pain (2 cases), liver dysfunction(1 case)	Abnormalities in liver and kidney function(4 cases)	LBS + CWM	CWM	
Niu et al. (2014) (41)	diarrhoe (6 cases)	diarrhoe (6 casse), spin (5 cases)insomnia (1 case)	LBS + CWM	CWM	
Zhu (2023) (42)	nauseating (2 cases), headaches (2 cases), diarrhoe (1 case), allergies (1 case)	nauseating (3 cases), headaches(2 cases), diarrhoe(1 case), allergies(1 case), hypotension (1 case)	LZP + CWM	CWM	
Zhao et al. (2023) (43)	pruritic (1 case), spin (1 case)	diarrhoe (1 case), nauseating(2 cases), spin(1 case)	LZP + CWM	CWM	
Li et al. (2022) (45)	0	diarrhoe (2 cases)	LZP + CWM	CWM	
Liang et al. (2020) (46)	skin rash (1 case), pruritic (1 case)nauseating (2 cases), diarrhoe (1 case)	skin rash (2 cases), nauseating(3 cases), diarrhoe(1 case)	XLQ + CWM	CWM	
Luo and Feng (2020) (47)	spin (1 case), nauseating (1 case)	spin (3 cases), headaches(2 cases), nauseating(2cases), diarrhoe(3 cases)	XLQ + CWM	CWM	
Yang and Wang (2019) (49)	0	0	XLQ + CWM	CWM	
Li et al. (2020) (50)	0	0	NMT + CWM	CWM	
Ye (2019) (52)	spin (1 case), tinnitus (1 case), fatigue (2 cases)	spin (2 cases), tinnitus (2 cases), fatigue (2 cases), skin rash (1 case)	NMT + CWM	CWM	
Deng et al. (2018) (53)	spin (1 case), diarrhoe (3 cases)	headaches (2 cases), spin (3 cases), hypotension (3 cases)	NMT + CWM	CWM	
Zhao et al. (2020) (54)	0	0	QLX + CWM	CWM	
Xu et al. (2020) (55)	spin (3 cases), diarrhoe (2 cases)	0	QLX + CWM	CWM	
Fan et al. (2023) (58)	nauseating (2 cases), skin rash (2 cases), ejaculatory abnormality (1 case), pruritic (3 cases), testicular pain (1 cases)	nauseating (1 case), skin rash(1 case), ejaculatory abnormality(2 cases), pruritic(1 case), testicular pain (1 case)	QLSL+CWM	CWM	
Cui et al. (2020) (60)	0	spin (1 case)	QLSL+CWM	CWM	
Gu (2021) (61)	0	0	WLT + CWM	CWM	
Jiang and Wu (2019) (62)	3 cases	4 cases	WLT + CWM	CWM	
Xiang and Xiao (2012) (64)	diarrhoe (3 cases)	spin (4 cases)	ZGLS+CWM	CWM	
Li et al. (2009) (66)	spin (2 cases), diarrhoe (1 case)	spin (1 case)	ZGLS+CWM	CWM	
Zhang et al. (2020) (67)	Hypotension (1 case), arteriosclerosis (1 case), skin rash (1 case)	Hypotension (1 case), arteriosclerosis (1 case), skin rash (1 case)	QLS + CWM	CWM	
Chang et al. (2020) (68)	diarrhoe (1 case), tachycardia (1 case), spin (2 cases), skin rash (1 case)	diarrhoe (1 case), tachycardia(1 case), spin (2 cases)	QLS + CWM	CWM	
Sun et al. (2019) (70)	spin (1 case), diarrhoe (1 case), pruritic (1 case)	headaches (2cases), tachycardia (2 cases), diarrhoe (3 cases), pruritic (2 cases)	HE+CWM	CWM	
Xu et al. (2021) (71)	drowsiness (1 case)	drowsiness (1 case), impotence (1 case), spin (1 cases)	JKSQ+CWM	CWM	
Shen (2018) (73)	spin (1 cases), impotence (1 case), skin rash (2 cases)	spin (2 cases), impotence(2cases), skin rash (1 case), diarrhoe (1 case)	JKSQ+CWM	CWM	
Kong et al. (2020) (75)	spin (2 cases), headaches(2cases), skin rash (1 case), diarrhoe (2 cases)	spin (2 cases), headaches(3 cases), diarrhoe (2 cases)	PLA + CWM	CWM	
Li (2012) (76)	spin (2 cases), diarrhoe (1 case)	spin (2 cases)	PLA + CWM	CWM	
Su (2012) (77)	spin (1 case)	nauseating (1 case)	PLA + CWM	CWM	
Ma et al. (2020) (78)	headaches (2cases), spin (2 cases), abdominal pain (2 cases)	headaches (3cases), spin (3 cases), abdominal pain (2 cases)	QLJD+CWM	CWM	
Zhang (2014) (79)	0	0	QLJD+CWM	CWM	
Yuan and Luo (2012) (81)	0	0	RLQ + CWM	CWM	

Compared with CWM alone, LBS + CWM (MD 0.59, 95% CI 0.41–0.85, very low certainty), NMT + CWM (MD 0.29, 95% CI 0.10–0.83, low certainty), HE + CWM (MD 3.76, 95% CI 1.74–8.14, low certainty) were beneficial to reduce adverse reactions (Table 9). Compared with CWM alone, there were no significant differences in others in the treatment of BPH (Table 9).

**Table 9:** Results of the network meta-analysis of the adverse reactions.

															
CWM															
0.88 (0.58,1.35)	QLST+CWM														
0.59 (0.41,0.85)	0.67 (0.39,1.18)	LBS + CWM													
0.61 (0.24,1.56)	0.69 (0.25,1.94)	1.03 (0.38,2.81)	LZP + CWM												
0.64 (0.24,1.69)	0.73 (0.25,2.10)	1.08 (0.38,3.04)	1.05 (0.27,4.06)	XLQ + CWM											
0.29 (0.10,0.83)	0.33 (0.10,1.03)	0.49 (0.16,1.49)	0.47 (0.11,1.95)	0.45 (0.11,1.90)	NMT + CWM										
0.56 (0.19,1.67)	0.63 (0.20,2.05)	0.94 (0.30,2.99)	0.92 (0.22,3.88)	0.87 (0.20,3.78)	1.94 (0.42,8.94)	QLX + CWM									
1.17 (0.42,3.25)	1.33 (0.44,4.01)	1.97 (0.67,5.82)	1.92 (0.48,7.68)	1.83 (0.45,7.48)	4.06 (0.93,17.73)	2.09 (0.47,9.36)	QLSL+CWM								
0.77 (0.19,3.19)	0.88 (0.20,3.85)	1.30 (0.30,5.61)	1.27 (0.23,6.93)	1.21 (0.22,6.73)	2.68 (0.46,15.77)	1.38 (0.23,8.29)	0.66 (0.12,3.79)	WLT + CWM							
0.93 (0.26,3.38)	1.06 (0.27,4.10)	1.57 (0.41,5.97)	1.53 (0.31,7.52)	1.46 (0.29,7.31)	3.24 (0.61,17.18)	1.67 (0.31,9.04)	0.80 (0.15,4.12)	1.21 (0.18,8.19)	ZGLS+CWM						
1.56 (0.53,4.58)	1.76 (0.55,5.63)	2.62 (0.84,8.18)	2.55 (0.61,10.67)	2.43 (0.57,10.38)	5.40 (1.19,24.56)	2.78 (0.60,12.96)	1.33 (0.30,5.88)	2.01 (0.34,11.96)	1.67 (0.31,8.93)	QLS + CWM					
0.26 (0.07,0.99)	0.29 (0.07,1.20)	0.43 (0.11,1.75)	0.42 (0.08,2.18)	0.40 (0.08,2.12)	0.89 (0.16,4.97)	0.46 (0.08,2.61)	0.22 (0.04,1.19)	0.33 (0.05,2.35)	0.27 (0.04,1.77)	0.16 (0.03,0.93)	HE+CWM				
1.05 (0.33,3.36)	1.19 (0.35,4.11)	1.77 (0.52,5.98)	1.72 (0.39,7.68)	1.64 (0.36,7.47)	3.65 (0.75,17.63)	1.88 (0.38,9.29)	0.90 (0.19,4.23)	1.36 (0.22,8.51)	1.13 (0.20,6.37)	0.68 (0.14,3.30)	4.12 (0.69,24.60)	JKSQ+CWM			
0.99 (0.39,2.53)	1.13 (0.40,3.14)	1.67 (0.61,4.55)	1.63 (0.43,6.13)	1.55 (0.40,5.97)	3.45 (0.84,14.20)	1.78 (0.42,7.50)	0.85 (0.21,3.39)	1.29 (0.24,7.02)	1.06 (0.22,5.21)	0.64 (0.15,2.66)	3.89 (0.75,20.22)	0.94 (0.21,4.20)	PLA + CWM		
0.86 (0.30,2.42)	0.97 (0.32,2.98)	1.44 (0.48,4.33)	1.40 (0.35,5.69)	1.34 (0.32,5.54)	2.97 (0.67,13.13)	1.53 (0.34,6.93)	0.73 (0.17,3.14)	1.11 (0.19,6.42)	0.92 (0.18,4.78)	0.55 (0.12,2.46)	3.35 (0.61,18.52)	0.81 (0.17,3.87)	0.86 (0.21,3.48)	QLJD+CWM	
0.78 (0.02,40.09)	0.88 (0.02,46.50)	1.31 (0.02,68.60)	1.27 (0.02,73.38)	1.21 (0.02,70.45)	2.70 (0.05,160.13)	1.39 (0.02,83.25)	0.66 (0.01,39.04)	1.01 (0.02,66.43)	0.83 (0.01,52.67)	0.50 (0.01,29.80)	3.05 (0.05,197.06)	0.74 (0.01,45.09)	0.78 (0.01,45.02)	0.91 (0.02,53.60)	RLQ + CWM

Based on the SUCRA values, HE + CWM had the highest probability of reducing adverse reactions (SUCRA: 87.2%), followed by NMT + CWM (SUCRA: 87%), and LBS + CWM (SUCRA: 65.9%; Figure 5F; Table 3).

### 3.5 Cluster analysis

**Figure 6:**
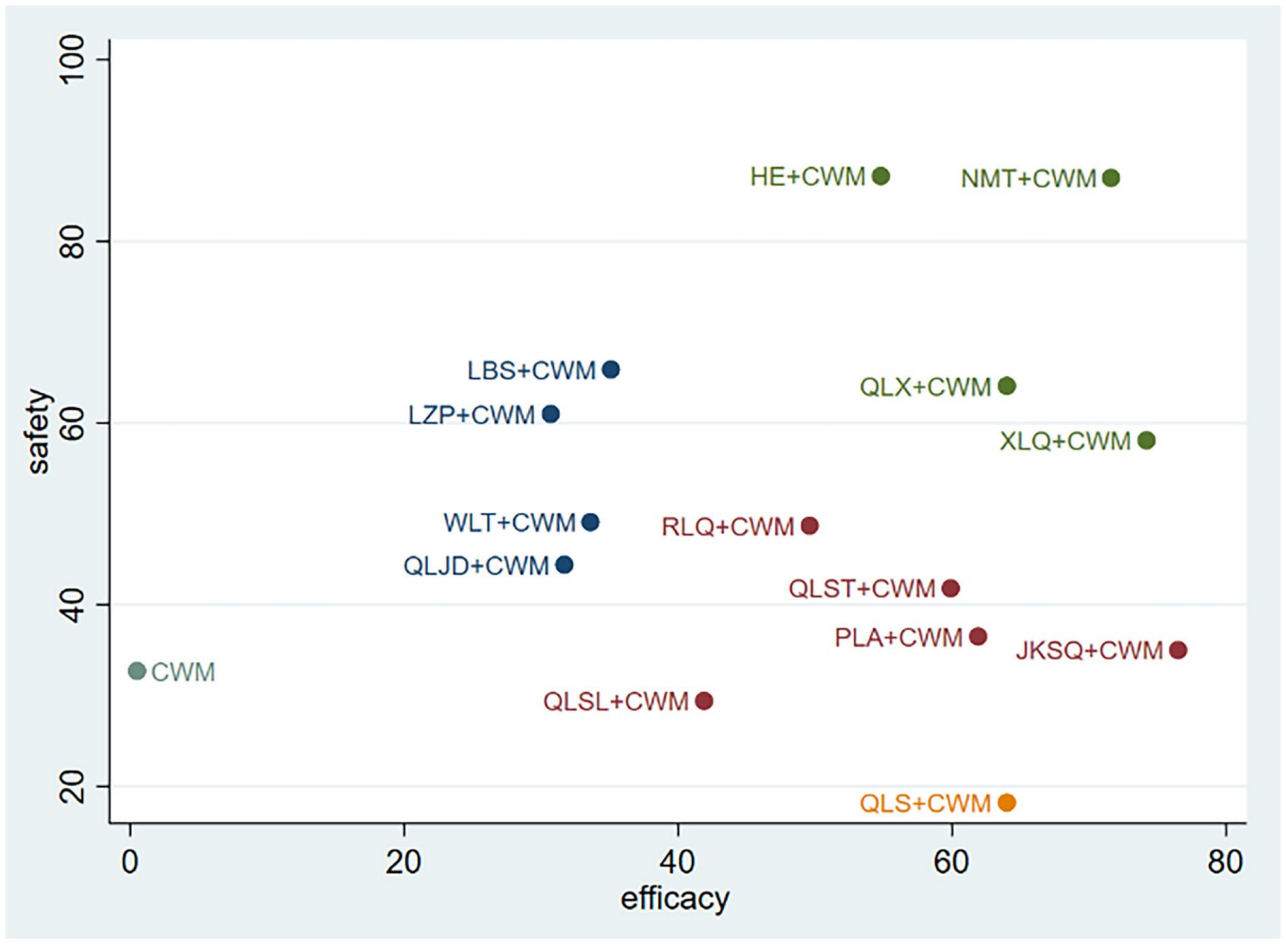
Cluster analysis plots. Interventions located in the upper right corner indicate optimal combination therapy for two different outcomes.

The impact of intervention measures on two different outcomes was comprehensively compared through cluster analysis. The study conducted two cluster analyses, including the clinical efficacy and therapeutic effect of BPH. The results are shown in Figure 6. Through comprehensive analysis of cluster analysis, JKSQ + WM has better efficacy and HE + WM has higher in safety. Considering the combination of effectiveness and safety, NMT + CWM might possess good therapeutic results and high safety.

### 3.6 Inconsistency test

There is a lack of direct comparison among various intervention measures, and no closed loop has been found in NMA. We are unable to conduct inconsistency testing. Therefore, a consistency model for further analysis is used.

### 3.7 Publication bias

**Figure 7:**
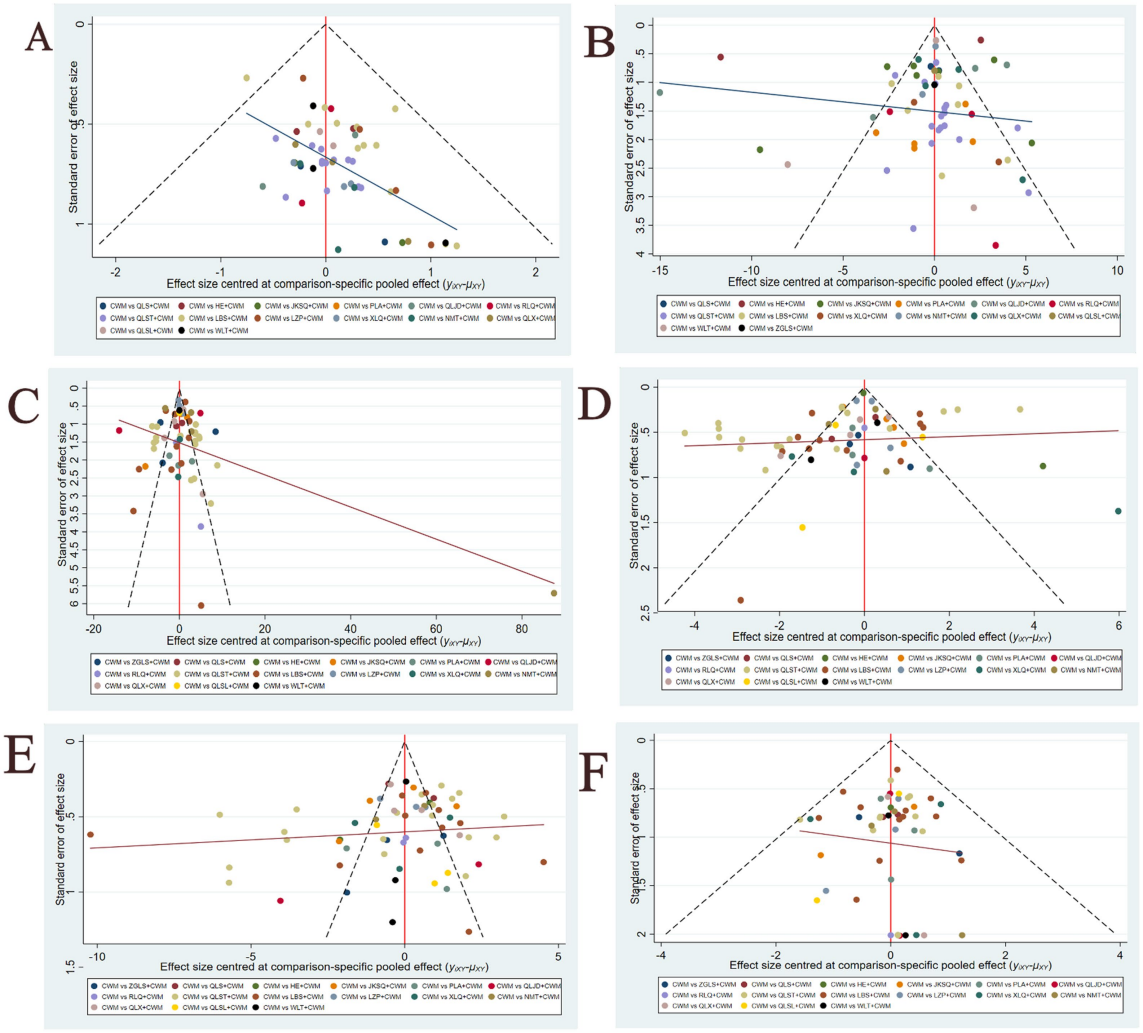
Funnel plots**(A)** Total efficiency ratio; **(B)** prostate volume; **(C)** residure volume; **(D)** maximum urinary flow rate; **(E)** international prostate symptom score; **(F)** adverse reactions.

Figure 7 shows a funnel plot of six main results to assess publication bias. All funnel plots are not completely symmetrical visually, and each adjusted auxiliary line is not perpendicular to the centerline. Therefore, there may be significant publishing deviations.

## 4 Discussion

### 4.1 Summary of findings

This research systematically evaluates the efficacy of 15 frequently utilized OCPMs (QLST, LBS, LZP, XLQ, NMT, QLX, QLSL, WLT, ZGLS, QLSW, HE, JKSQ, PLA, QLJD, RLQ) in conjunction with CWMs for the treatment of BPH, based on data from 72 relevant studies employing network meta-analysis. The findings from the NMA indicate that the majority of OCPMs in conjunction with CWM outperformed CWM alone across all outcomes, with statistically significant differences observed between the groups.

Considering the statistical variability and SUCRA results, in total efficiency ratio, JKSQ + CWM was most likely to be the best treatment option; in prostate volume, HE + CWM was most likely to be the best treatment option; in residure volume, QLST + CWM was most likely to be the optimal treatment regimen; in maximum urinary flow rate, XLQ + CWM was most likely to be the optimal treatment regimen; in IPSS, LBS + CWM was most likely to be the optimal treatment regimen; in adverse reactions, HE + CWM was most likely to be the optimal treatment regimen.

Combining the two indicators of total efficiency ratio and adverse reactions, NMT + CWM is the best treatment option for BPH. Therefore, the efficacy of NMT + CWM in treating BPH is worthy of attention, but clinicians should also choose the appropriate method according to the specific conditions of clinical patients.

### 4.2 Research significance and importance

The precise process of benign prostatic hyperplasia (BPH) remains unclear; it may result from the regulated destruction of epithelial and mesenchymal cell proliferation and death. Its etiology also encompasses the interplay of androgens, estrogens, growth hormones, and inflammatory cells, among other factors. In Chinese medicine, it is classified under “essence retention.” In Chinese medicine, this ailment is classified as “urinary retention” and predominantly affects elderly men with deficiencies in the spleen and kidneys, resulting in bladder failure, water-dampness, and the accumulation of damp-heat in the lower body, which contributes to the condition.

Alpha blockers can alleviate lower urinary tract symptoms and pain by relaxing the smooth muscle of the prostate and bladder, while 5α-reductase inhibitors can diminish prostate size (7). According to Chinese medicine theory, prostate hyperplasia is associated with deficiency and stasis; oral Chinese patent medicines can address spleen and kidney deficiency, enhance kidney function and blood circulation, and regulate bladder urinary storage capacity. Consequently, oral Chinese patent medicines and standard Western treatments are complementary and equally significant.

Oral Chinese patent medicines are offered in tablets, pills, capsules, granules, powders, and oral liquids, providing advantages such as convenient clinical use, ease of storage and transportation, and excellent safety, making them widely utilized in the treatment of BPH (82). Currently, a diverse array of oral Chinese patent medicines is employed for the management of BPH. Nevertheless, there is an absence of cross-sectional comparisons among the various medications. This study involved a retrospective meta-analysis utilizing a frequency-based methodology to evaluate the efficacy and safety of various OCPMs and to rank their respective advantages and disadvantages.

### 4.3 Limitations

The quality of the included studies was inconsistent; several studies failed to specify the randomization method, potentially introducing random error. Furthermore, the majority of studies did not detail the execution of allocation concealment and blinding, which could lead to selection bias and affect the validity of the study results.

The quantity of included studies exhibited considerable variability among OCPMs, potentially influencing the conclusions. The absence of direct comparisons among OCPMs may have affected the dependability of the results.

### 4.4 Prospects

We hereby present the subsequent recommendations. The study’s inclusion criteria are inadequate in rigor in identification and classification, with the application of OCPMs reliant on symptom identification guided by traditional Chinese medicine philosophy. Consequently, we recommend that clinicians select the OCPMs with optimal therapeutic efficacy aligned with the patient’s symptoms, contingent upon precise diagnosis, to enhance treatment effectiveness, decrease disease duration, and alleviate patient discomfort. Secondly, in the execution of randomized controlled trials, multicenter randomized double-blind trials must be conducted in strict compliance with pertinent rules, and meticulous attention should be given to the random number generation, allocation concealment, and blinding procedures. Finally, multicenter large-sample randomized controlled RCTs need to be conducted to compare the effects of different OCPMs to make up for the lack of research in this area. Implementing the study across various centers can enhance the control of potential confounders and selection biases, so as to augment the study’s authenticity. Furthermore, boosting the sample size can bolster the study’s reliability.

## 5 Conclusion

The efficacy of OCPM combined with CWM in the treatment of BPH is better than that of CWM alone, and the safety is good; various OCPMs have different therapeutic focuses, which can be individualized according to the specific symptoms of BPH patients in the clinic. Moreover, with the combined efficacy and safety, NMT + CWM is worth paying attention to in CPM. However, considering the limitations of this paper, these conclusions should be verified by multicenter, high-quality, large-sample randomized controlled trials.

## Data Availability

The original contributions presented in the study are included in the article/Supplementary material, further inquiries can be directed to the corresponding authors.
