# Supplementary material for: Efficacy and safety of oral Chinese patent medicine for benign prostatic hyperplasia: a network meta-analysis of randomized controlled trials
Source: Front Med (Lausanne). 2024 Dec 27;11:1483864. doi: 10.3389/fmed.2024.1483864 (PMC11718654; doi:10.3389/fmed.2024.1483864)
Supplement: Supplementary file 1 [file Table_1.DOCX]

#1(Benign prostatic hypertrophy[MeSHT Terms]) OR (prostate hypertrophy [MeSHT Terms]) OR (Benign prostatic hypertrophy [Title/Abstract]) OR (prostate hypertrophy [Title/Abstract]) OR (prostate enlargement [Title/Abstract])

#2(Chinese medicine [MeSHT Terms]) OR (Chinese patent medicine [MeSHT Terms]) OR (Chinese medicine [Title/Abstract]) OR (Chinese patent medicine [Title/Abstract]) OR (capsule [Title/Abstract]) OR pellet [Title/Abstract]) OR (powder [Title/Abstract]) OR (pill [Title/Abstract])

#3(overall effectiveness rate [MeSHT Terms]) OR (International Prostate Symptom Score [MeSHT Terms]) OR (Postvoid Residual Volume [MeSHT Terms]) OR (Prostate Volume [MeSHT Terms]) OR (mean maximal flow rate [MeSHT Terms]) OR (overall effectiveness rate [Title/Abstract]) OR (International Prostate Symptom Score [Title/Abstract]) OR (Postvoid Residual Volume [Title/Abstract]) OR (Prostate Volume [Title/Abstract]) OR (mean maximal flow rate[Title/Abstract])

#4 (randomized controlled trial [Publication Type]) OR (controlled clinical trial [Publication Type])

OR (randomization [Title/Abstract]) OR (randomized [Title/Abstract]) OR (RCT [Title/Abstract]) OR (trial [Title/Abstract])

#5("1000/1/1"[Date-Publication]:"2023/11/31"[Date-Publication])

#6 #1 AND #2 AND #3 AND #4 AND #5
